# Hydrologic Variability Governs Population Dynamics of a Vulnerable Amphibian in an Arid Environment

**DOI:** 10.1371/journal.pone.0125670

**Published:** 2015-06-01

**Authors:** Erin R. Zylstra, Robert J. Steidl, Don E. Swann, Kristina Ratzlaff

**Affiliations:** 1 School of Natural Resources and the Environment, University of Arizona, Tucson, Arizona, United States of America; 2 Saguaro National Park, Tucson, Arizona, United States of America; University of South Dakota, UNITED STATES

## Abstract

Dynamics of many amphibian populations are governed by the distribution and availability of water. Therefore, understanding the hydrological mechanisms that explain spatial and temporal variation in occupancy and abundance will improve our ability to conserve and recover populations of vulnerable amphibians. We used 16 years of survey data from intermittent mountain streams in the Sonoran Desert to evaluate how availability of surface water affected survival and adult recruitment of a threatened amphibian, the lowland leopard frog (*Lithobates yavapaiensis*). Across the entire study period, monthly survival of adults ranged from 0.72 to 0.99 during summer and 0.59 to 0.94 during winter and increased with availability of surface water (*Z* = 7.66; *P* < 0.01). Recruitment of frogs into the adult age class occurred primarily during winter and ranged from 1.9 to 3.8 individuals/season/pool; like survival, recruitment increased with availability of surface water (*Z* = 3.67; *P* < 0.01). Although abundance of frogs varied across seasons and years, we found no evidence of a systematic trend during the 16-year study period. Given the strong influence of surface water on population dynamics of leopard frogs, conservation of many riparian obligates in this and similar arid regions likely depends critically on minimizing threats to structures and ecosystem processes that maintain surface waters. Understanding the influence of surface-water availability on riparian organisms is particularly important because climate change is likely to decrease precipitation and increase ambient temperatures in desert riparian systems, both of which have the potential to alter fundamentally the hydrology of these systems.

## Introduction

Climate is one of the principal forces that governs the distribution and demography of animal populations (e.g., [1–3]). In particular, temperature and precipitation regimes define distributional limits for many species [4] and explain variation in demographic rates within and among populations (e.g., [3, 5–6]). Effects of climate may be especially influential for species with limited abilities to shift their geographic ranges in response to anticipated future changes in climate [7–8]. For these species, identifying the primary mechanisms that govern interactions between climate and demography will be essential to understanding the natural dynamics of their populations and for developing effective conservation strategies.

Mobility of many species that inhabit aquatic ecosystems in arid environments is limited, which will constrain their ability to shift their geographic distributions in response to climate change. Further, because the aquatic resources on which these species rely are isolated, connectivity among habitat patches is low. In the southwestern U.S., these rare aquatic ecosystems support an unusually diverse array of taxa that depend on surface water for some aspect of their life history [9–11]. Therefore, maintaining the ecological structure and functions of these ecosystems is essential to maintaining biodiversity throughout the region. This may become increasingly difficult, however, as precipitation and snowpack are predicted to decrease [12–13] and the frequency of large wildfires, which are often associated with sedimentation events in mountain canyons, are predicted to increase [14]. All of these changes have potential to decrease availability of aquatic resources in a region that has experienced increased drought conditions over the past 30 years [15].

In arid regions, the influence of climate on stream flow, vegetation, and vertebrates has been relatively well-studied in high-order, valley-bottom streams (e.g., [16–18]). In contrast, effects of climate on vertebrates that inhabit low-order streams in these regions have been studied less. In most of these stream systems, flow is intermittent and a product of both surface runoff and groundwater [19], which creates patches of surface water that are highly dynamic in space and time. Fundamental shifts in the availability of these aquatic resources could have significant conservation implications for species that are aquatic obligates, ultimately affecting their distribution, demography, and phenology.

One such species, the lowland leopard frog (*Lithobates yavapaiensis*), is distributed across the southwestern U.S. and northwestern Mexico. The historical distribution of the species likely included perennial stream systems that coursed valley bottoms, but has since contracted to low-order intermittent streams as flow in nearly all valley-bottom waterways has declined from perennial to ephemeral [16], [19–23]. As a consequence of reductions in habitat and other threats that include disease and invasions by nonnative species, lowland leopard frogs have been designated as a “species of greatest conservation need” in Arizona [20–21], [24–25]. Although the species seems to be declining throughout the region, and *Batrachochytrium dendrobatidis* (Bd)—the fungus that causes chytridiomycosis—has been associated with winter mortality [26–28], we know little about the long-term dynamics of these populations.

As is common for many amphibians, distribution and abundance of lowland leopard frogs has varied markedly over time and across space [21], [28–29]. Differentiating long-term declines that might lead to local extinctions from short-term natural fluctuations requires detailed field studies, especially for species such as lowland leopard frogs that inhabit variable environments [29]. Effective conservation relies on understanding factors that govern these population dynamics, which in turn requires data that span temporal and spatial scales that are sufficient to capture population fluctuations as well as variation in climate and other environmental features. These data can be especially challenging to acquire for species that are rare, difficult to detect, and expensive to survey.

To understand the influence of environmental features, particularly availability of surface water, on demography of lowland leopard frogs, we explored survey data collected over a 16-year period in mountain canyons of southern Arizona. For analysis, we employed hierarchical models that allowed us to model variation in abundance from repeated count-based surveys while simultaneously accounting for processes affecting detection probability. Our findings will help to inform long-term conservation strategies for lowland leopard frogs and other riparian obligates that depend on surface water in arid ecosystems and that are likely to be affected by climate change.

## Materials and Methods

### Study area

We studied lowland leopard frogs in the Madrean Sky Islands, a region that spans the southwestern U.S. and northern Mexico, where isolated mountain ranges are surrounded by vast expanses of desert and semi-desert grassland. Specifically, we surveyed frogs throughout the Rincon Mountains of Saguaro National Park in southern Arizona. Vegetation composition varies with elevation, ranging from Sonoran desertscrub at the lowest elevations (900 m) to pine and mixed-conifer forests at the highest elevations (2640 m) [30]. Average annual precipitation also varies with elevation, ranging from 330 mm to >700 mm, and is seasonally bimodal, with high-intensity monsoonal storms in July and August and low-intensity winter rains between November and March [30]. The mountain range is bisected by a ridge formation, with deeply-incised canyons north of the ridge forming part of one watershed and canyons west and south of the ridge forming part of a second watershed. Pools occur within deeper canyons and are often connected by shallow riffles and runs during wet periods (late winter [Dec-April] and summer [Jul-Sep]) but separated by dry channels when areas between pools dry seasonally [19], [22].

In the Sky Island region, lowland leopard frogs inhabit pools along low-order, intermittent streams below approximately 1400 m elevation. In 1996, all major canyons in Saguaro National Park were surveyed during the driest part of the year (May-June) to inventory water resources and to identify potential habitat for lowland leopard frogs. More than 230 pools in seven canyons were classified as having potential to support lowland leopard frogs, including evidence of holding water for most of the year or supporting perennial aquatic vegetation, such as cattails (*Typha* spp.). Our analyses are based on a subset of 162 pools that were surveyed repeatedly between 1996 and 2011; these were distributed across four canyons, two in the north watershed and two in the south watershed.

### Field surveys

Availability of surface water in mountain canyons varies seasonally, which affects both frog activity and the ability of observers to detect frogs [19], [22]. Between 1996 and 2011, leopard frogs were surveyed during spring (1 May–15 July) and fall (1 October–30 November) when streams typically were not flowing; we refer to these periods as spring and fall sampling periods. We excluded surveys during summer (16 July–30 September) and winter (1 December–30 April) because survey effort was limited. Generally, streams were surveyed by one or two observers, at least one of which had extensive experience surveying for lowland leopard frogs in these mountain canyons. Observers followed standard protocols for visual encounter surveys during daylight hours [22], [31]; they used binoculars to survey the terrestrial perimeter of pools from a distance of 10–20 m or approached pools quietly to scan directly from a distance of <10 m. After an initial scan, observers approached pool edges and counted the number of adult lowland leopard frogs observed (approximate minimum snout-vent length of 55 mm) [21]. During surveys, observers recorded whether or not each pool contained water.

In addition to visual encounter surveys, we used mark-resight methods to characterize movements by adult lowland leopard frogs among pools in one canyon in the north watershed and one canyon in the south watershed. We were able to use mark-resight methods for these populations because (1) in the Sky Island region, lowland leopard frogs inhabit pools along linear stream segments that are surrounded by arid uplands that are inhospitable to aquatic species [21–22]; (2) unlike some ranids, post-metamorphic leopard frogs are thought to make short movements along watercourses and remain in close proximity to aquatic environments year round [21,23]; (3) breeding populations in mountain canyons are small relative to populations of many other anurans [23], [32]. We surveyed each canyon approximately once per week between May and December 2013 and used photographs taken in situ to identify individuals by their unique dorsal and lateral spot patterns [33]. We only classified observations as resightings if we obtained photographic confirmation of matching spot patterns from ≥2 locations on the dorsal and lateral surfaces of the frog. Frogs were never captured. Field permits were obtained from the National Park Service and the Arizona Game and Fish Department; the University of Arizona’s Institutional Animal Care and Use Committee approved protocols for the mark-resight portion of the project (Protocol number 13–450).

### Spatial structure of pools and temporal structure of surveys

Many pools were in close proximity and therefore did not function as independent habitat patches. Consequently, we classified sets of adjacent pools as “pool complexes” that we delineated based on the expectation that during periods when streams were not flowing, frogs were likely to remain at pools within one complex and not move among complexes. Therefore, we divided the 162 pools that were surveyed into 33 complexes, with each complex separated from the nearest adjacent complex by >120 m. We selected 120 m as the minimum distance between complexes because only 1 of 129 movements we documented during mark-resight surveys spanned a drainage segment >120 m when there was no flow or surface water present. We made no assumptions about movements of frogs during periods when surface water typically flows between pools.

Generally, we defined a survey as the set of observations of frogs and surface water from all pools in a complex visited in a single day. In larger canyons, however, surveys of complexes occasionally spanned two days within a seven-day period, which we considered a single survey. On average, each complex was surveyed during 19 of 32 sampling periods (60%). During those sampling periods when complexes were surveyed at least once, they were typically surveyed 1–3 times but occasionally 4–10 times (<4% of complex-sampling period combinations). To improve model convergence and speed computation times, we limited the number of surveys per sampling period by selecting a maximum of three surveys per sampling period and excluding surveys where the fewest number of pools were visited or the fewest frogs were observed.

### Environmental characteristics

We characterized the area of each canyon basin and the number of pools, elevation, and demographic connectivity of each pool complex. We used Automated Watershed Assessment Tools [34] in ArcGIS 10.0 to estimate the area of each canyon basin as total area measured from the lowest-elevation pool in each canyon. For each complex, we calculated elevation as the mean elevation across pools and quantified demographic connectivity, *S*_*i*_, with a dispersal kernel derived from a negative exponential function:

$$S_{i}=\underset{j\in W_{i}}{\sum }\exp\left(-\alpha d_{ij}\right),$$ (1)

where *W*_*i*_ is the set of all complexes surveyed within the same watershed as complex *i*; *d*_*ij*_ is stream distance in km between complex *i* and *j*; and α is a constant that scales the effect of distance to dispersal (1/α approximates average dispersal distance) [35]. We estimated α using the mark-resight data we collected. Specifically, we calculated the maximum linear distance moved along the stream for each frog in 2013, classified distances into 10-m intervals, and calculated the proportion of frogs that were likely to move at least as far as each of the distances that defined the lower limits of the 10-m intervals. We then estimated α by fitting these data to a negative-exponential function using the *nls* procedure in R [36]-[37]. Although these data are likely to underestimate dispersal probabilities over the lifespan of a typical frog, they represent the best data available for frogs in mountain-canyon systems. We used stream distances between complexes rather than Euclidean distances because areas outside of canyons are arid and inhospitable to frogs, and because genetic evidence suggests that lowland leopard frogs disperse primarily along canyon corridors (C. Goldberg, Washington State Univ., unpublished data). We did not limit neighboring complexes to those that were known to be inhabited during previous sampling periods because surveys were irregular and occupancy status could not be determined with certainty. Consequently, we refer to this measure as potential connectivity and assumed values were constant over time for each complex.

We characterized availability of surface water with two measures, one during surveys and one across seasons. To characterize variation in water availability during each survey (hereafter, survey-specific water availability), we calculated the proportion of pools with water in each complex. For 5.5% of surveys, observers neglected to record the number of pools with water, therefore we predicted values of water availability for those surveys with a logistic regression model that related the proportion of pools with water in each complex in spring or fall to precipitation totals during the previous winter or summer, respectively. Because precipitation data were not available for specific canyons, we used daily precipitation data from an Arizona Meteorological Network weather station in Tucson, AZ located approximately 25 km from our study area. To assess variation in surface water across seasons (hereafter, seasonal water availability), we calculated the minimum proportion of pools with water across all surveys completed during each spring and fall sampling period (i.e., minimum value of survey-specific water availability during each sampling period). Seasonal water availability during a spring or fall sampling period likely reflected the minimum amount of surface water available during the subsequent summer or winter non-sampling period, respectively, because precipitation totals and flow of surface water are typically higher for summer and winter seasons than spring and fall [19].

### Analyses

We used a hierarchical framework that allowed us to account for imperfect surveys to model the dynamics of adult leopard frog abundances over the 32 sampling periods between 1996 and 2011. We used counts of the number of adult frogs summed across all pools in a complex during each survey to estimate four parameters: abundance in the first sampling period (hereafter, initial abundance; *λ*_1_), recruitment of individuals into the adult stage class (γ), apparent survival (ω), and detection probability (*p*) [38]. With this approach, we assume demographic and geographic closure within sampling periods, but allow abundance to vary between sampling periods as a function of recruitment and survival, the dynamic parameters of primary interest. Recruitment includes juveniles that are recruited into the adult stage class and adults that immigrate from other complexes. Apparent survival (hereafter, survival) describes the probability that adults survive and remain in a complex until the next sampling period. We assumed no relationship between recruitment and survival and allowed recruitment to vary independently of abundance in the previous sampling period.

We started with a fully parameterized (global) model that included all environmental and survey characteristics that we hypothesized could explain variation in initial abundance (number of pools per complex, canyon-basin area, elevation), recruitment (season, seasonal water availability, number of pools per complex, connectivity), survival (season, seasonal water availability, number of pools per complex), and detection probability (survey-specific water availability, date, survey effort) of lowland leopard frogs. We used a zero-inflated Poisson distribution to describe initial abundance because adult leopard frogs were absent or undetected at complexes during 61% of surveys and because this distribution was less likely to inflate estimates of initial abundance than a negative binomial distribution [39]. We used log-transformed number of pools per complex as an offset for initial abundance because it allowed us to account for variation in the amount of habitat per complex, which is likely to affect abundance. In addition to the offset, we allowed initial abundance to vary with area of canyon basin and mean elevation. Because we anticipated that initial abundance may be highest at intermediate elevations, we included a quadratic term for mean elevation in the global model. We allowed both recruitment and survival to vary with season (summer or winter), seasonal water availability, and number of pools per complex. We also allowed recruitment to vary as a function of connectivity because proximity of neighboring complexes could explain variation in the number of adults immigrating into a complex. We anticipated that detection probability could vary with survey-specific water availability, date, and survey effort, which we measured as the proportion of pools in each complex that were visited by observers during each survey. We modeled variation in detection probability as a linear function of survey effort. We were less certain of how detection probability might vary with survey-specific water availability or date, so we included terms in the global model to allow for non-linear relationships. Specifically, we included a quadratic term for survey-specific water availability and allowed detection probability to vary as a quadratic function of date (day number within season) independently within spring and fall sampling periods (day + day^2^ + season + season*day + season*day^2^).

We used a step-down approach to identify a final model for inference by retaining only those environmental and survey characteristics from the global model that explained appreciable variation in each parameter (*P* < 0.05 based on partial *Z*-tests). For characteristics with significant interaction or quadratic terms, we retained their constituent main effects in the final model. We standardized variables that were not proportions prior to analysis and implemented all models with the R package *unmarked* [40]. To evaluate temporal trends in overall abundance, we used empirical Bayes methods to estimate mean abundance of frogs in each complex in each of the 32 seasons [41]. We then summed estimates of abundance across all complexes within each season. We fit a generalized linear model with a first-order autoregressive covariance structure to assess whether abundance changed linearly over the study period; we included season in the model to account for differences in abundance between spring and fall sampling periods. We report estimates ± 1 SE unless stated otherwise.

## Results

We identified 33 complexes in 4 canyons, each of which was comprised of between 1 and 17 pools (mean = 4.9 ± 0.75), with distances between adjacent complexes in the same watershed ranging from 123 to 11361 m (median = 3161 m). Complexes ranged in elevation from 849 to 1313 m and basin area of canyons ranged from 10.1 to 32.9 km^2^ (Table 1). Across all complexes and seasons, survey-specific water availability averaged 0.63 ± 0.015 but sometimes varied markedly within sampling periods. For example, within one sampling period at a single complex, water availability ranged from 0 to 1 on 17 different occasions. Seasonal water availability was also highly dynamic, both spatially and temporally. Averaged across sampling periods, seasonal water availability ranged from 0.25 to 1.00 among complexes (*n* = 33); averaged across complexes, seasonal water availability ranged from 0.30 to 0.95 among sampling periods (*n* = 32).

**Table 1:** Mean, standard error (SE), and range for site-, season-, and survey-specific characteristics used to explain variation in initial abundance, recruitment, survival, and detection probability of adult lowland leopard frogs between 1996 and 2011 in the Rincon Mountains, Arizona, USA.

Level of organization	Characteristic	*n*	Mean	SE	Range
Site	No. pools/complex ^a^	33	4.9	0.75	1 – 17
	Elevation (m) ^a^	33	1035.7	22.09	849–1313
	Canyon-basin area (km^2^) ^a^	4	18.47	5.354	10.1 – 32.9
	Connectivity (*S*_*i*_) ^a^	33	0.24	0.020	0.00004 – 0.44
Season	Seasonal water availability ^b^	602	0.62	0.017	0 – 1
Survey	Survey-specific water availability ^b^	811	0.63	0.015	0–1
	Effort	858	0.93	0.006	0.06 – 1

^a^ Values were standardized relative to their means and standard deviations for analysis.

^b^ Includes only data based on field observations.

Across the 16-year survey period, 33 pool complexes were surveyed 500 times during spring and 358 times during fall sampling periods. On average, each complex was surveyed during 19.2 ± 1.04 of 32 possible sampling periods (range = 3–28) and 1.4 ± 0.02 times per period during those sampling periods when the complex was surveyed at least once. Adult leopard frogs were observed at least once in 29 of 33 complexes (88%). The number of adult lowland leopard frogs observed in each complex during each survey ranged from 0 to 72, with more frogs observed in fall (5.1 ± 0.62 adult frogs/complex/survey) than in spring (1.6 ± 0.20).

We identified 105 unique adult frogs between May and December 2013, 90% (*n* = 95) of which we resighted at least once. We documented 129 movements that ranged from 5–272 m along stream courses (mean = 63.6 ± 4.68), 5% (*n* = 7) of which were between complexes. Of these seven inter-complex movements, only one occurred when there was no surface water between complexes. For frogs resighted at >1 pool (*n* = 67), the maximum distance moved ranged from 13–278 m (89.5 ± 7.82). Potential connectivity ranged from <0.01 to 0.44 among complexes based on an estimated α of 10.38.

**Fig 1:**
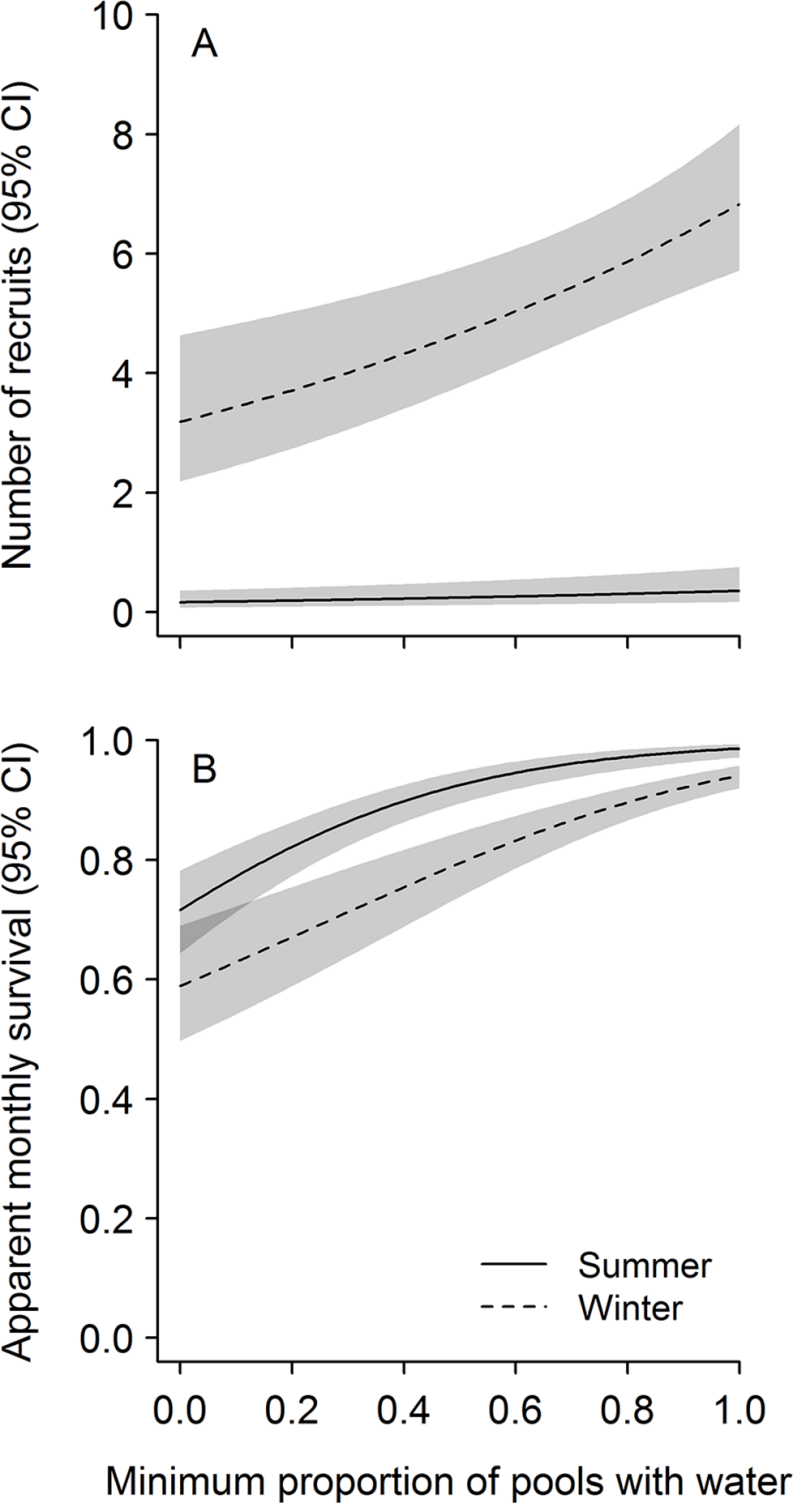
Predicted number of recruits and monthly survival of adult lowland leopard frogs as a function of seasonal water availability between 1996 and 2011 in the Rincon Mountains, Arizona, USA. Predicted number of recruits into the adult stage class for an average-sized pool complex (A) and monthly survival (B) with 95% confidence intervals. We made predictions from a model of recruitment as a function of season, seasonal water availability, number of pools per complex, and connectivity, and a model of survival as a function of season and seasonal water availability (Table 2). To predict the number of recruits, we held the number of pools per complex and connectivity values at the mean observed across all pool complexes (5 and 0.24, respectively).

**Fig 2:**
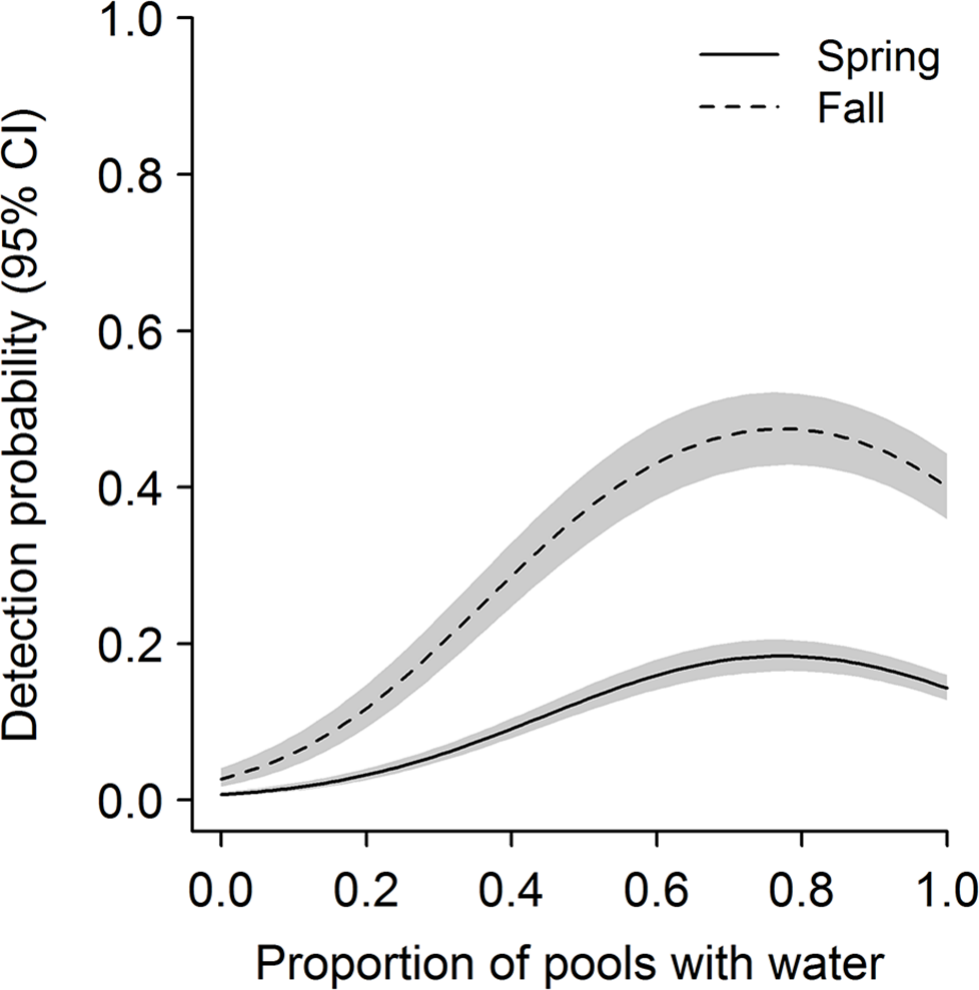
Detection probabilities of adult lowland leopard frogs as a function of survey-specific water availability between 1996 and 2011 in the Rincon Mountains, Arizona, USA. Shaded areas represent 95% confidence intervals. We made predictions from a model of detection probability as a function of effort, survey-specific water availability, and date (Table 2). We predicted detection probability for the midpoint of spring and fall sampling periods (approximately 7 June and 31 Oct) when observers surveyed pool complexes in their entirety (i.e., effort = 1).

**Fig 3:**
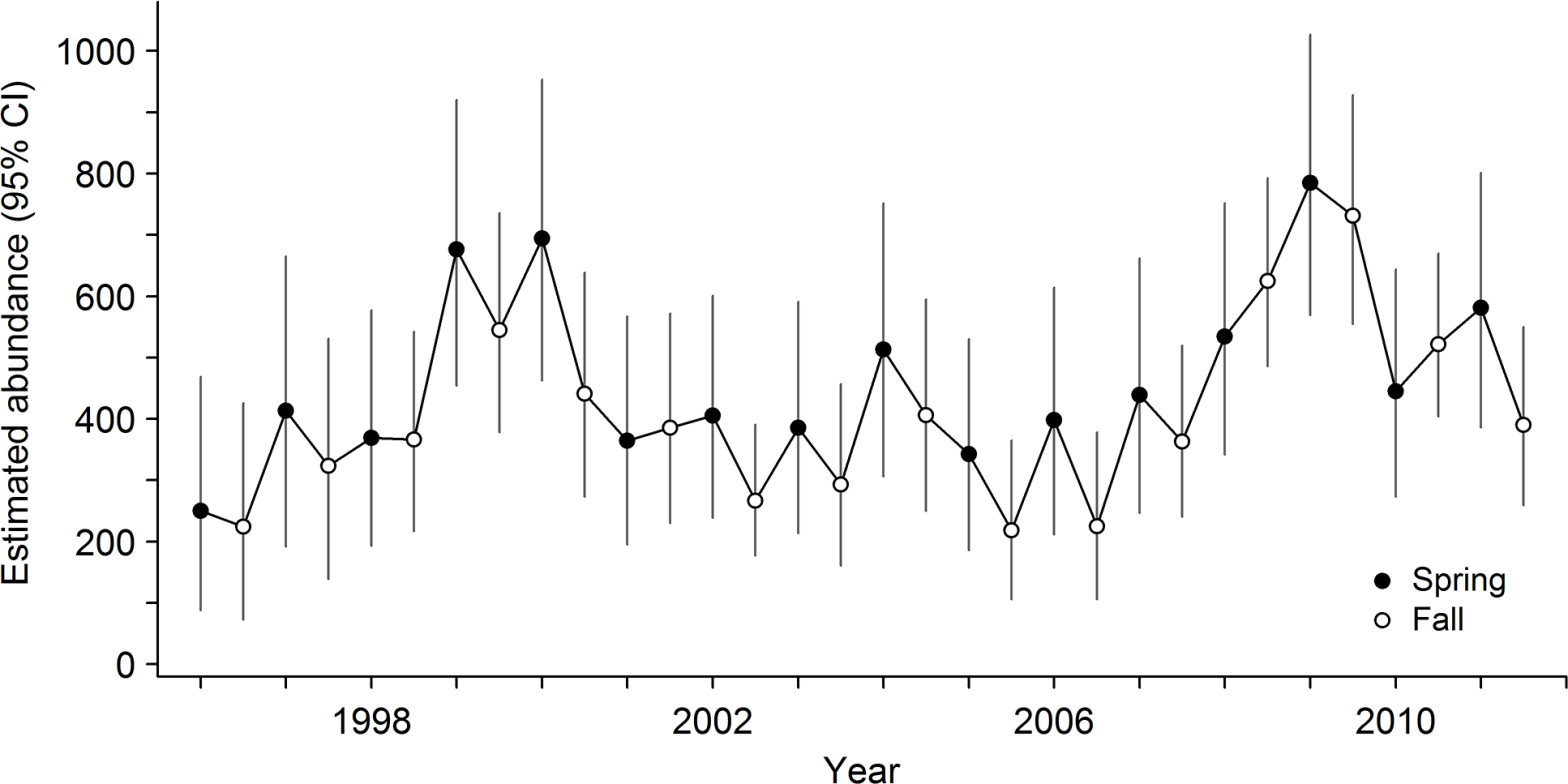
Estimated abundance of adult lowland leopard frogs in the Rincon Mountains, Arizona, USA between 1996 and 2011. Estimated number of adult lowland leopard frogs, with 95% confidence intervals, in four canyons in the Rincon Mountains during spring (1 May–15 July) and fall (1 October–30 November). Estimates were obtained using empirical Bayes methods based on a model of recruitment as a function of season, seasonal water availability, number of pools per complex, and connectivity, and a model of survival as a function of season and seasonal water availability (Table 2).

**Fig 4:**
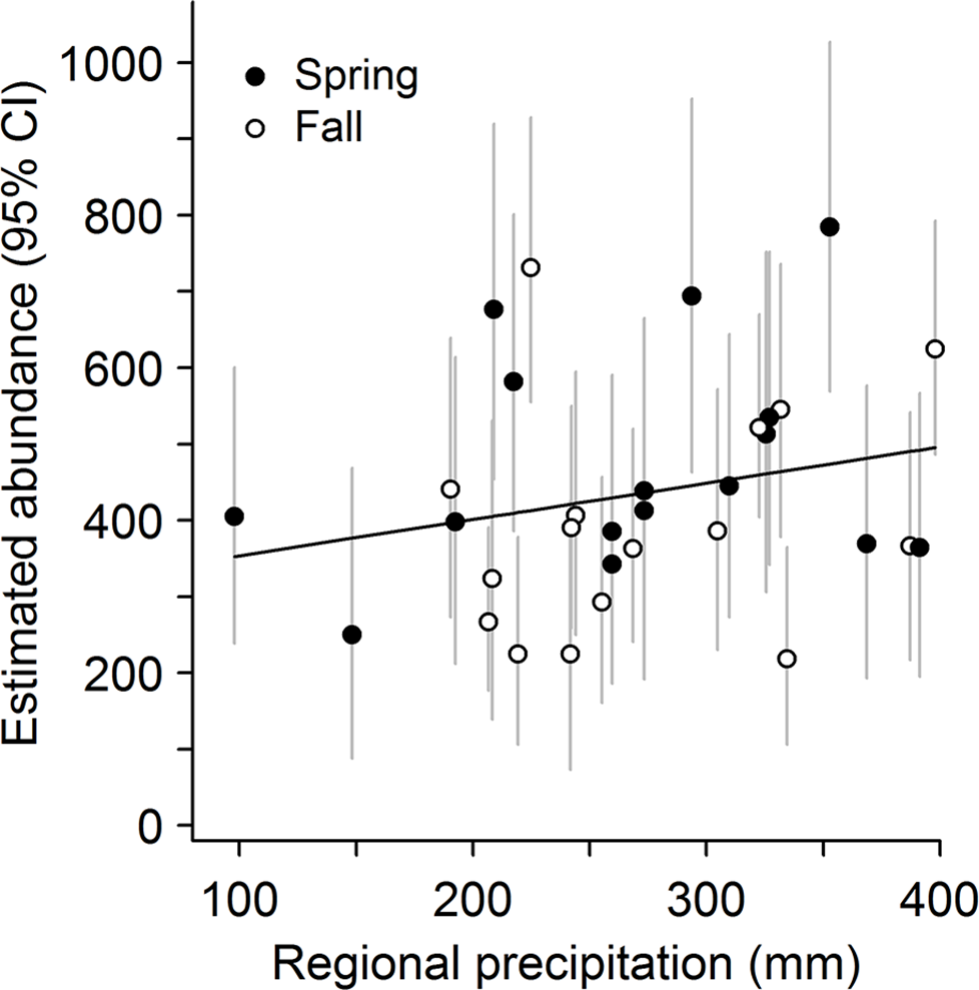
Estimated abundance of adult lowland leopard frogs in the Rincon Mountains, Arizona, USA between 1996 and 2011 as a function of regional precipitation for 12 months immediately preceding each sampling period. Estimated number of adult lowland leopard frogs, with 95% confidence intervals, in four canyons in the Rincon Mountains during spring (1 May–15 July) and fall (1 October–30 November). Estimates were obtained using empirical Bayes methods based on a model of recruitment as a function of season, seasonal water availability, number of pools per complex, and connectivity, and a model of survival as a function of season and seasonal water availability (Table 2). We obtained regional precipitation data from an Arizona Meteorological Network weather station in Tucson, AZ located approximately 25 km from our study area. For spring sampling periods, we summed monthly precipitation totals from May–April; for fall sampling periods, we summed monthly precipitation totals from October–September. The solid line represents a linear regression of abundance on precipitation.

Temporal variation in abundance of leopard frogs was governed primarily by variation in recruitment and survival within complexes; detection probability varied seasonally. Initial abundance—the estimated number of adult leopard frogs present during spring in the first year of the study—did not vary greatly among complexes, but was highest at intermediate elevations (highest predicted mean initial abundance for a complex with five pools = 23.5 adult frogs at 1099 m elevation) and did not vary with area of canyon basin (*Z* = 0.37, *P* = 0.71). Recruitment and survival of frogs varied over time and were governed, at least in part, by seasonal water availability. During summer, recruitment of frogs into the adult stage class was low, regardless of the amount of surface water available (Fig 1). During winter, however, mean recruitment in an average-sized complex ranged from 3.4 ± 0.59 adults when only 10% of pools contained water to 6.8 ± 0.62 adults when all pools contained water. Recruitment tended to increase as availability of surface water and number of pools per complex increased, and was higher at complexes that were more isolated (Table 2). Survival was higher in summer than in winter and increased with the amount of surface water in both seasons (Table 2, Fig 1); survival did not vary strongly with the number of pools per complex (*Z* = 1.64, *P* = 0.10). Across years, monthly survival ranged from 0.72 to 0.99 during summer (0.43–0.97 across a 2.5-month summer season) and 0.59 to 0.94 during winter (0.07–0.74 across a 5-month winter season). If we assume no mortality during sampling periods, annual adult survival was estimated to be 0.37 ± 0.05 when seasonal water availability equaled the mean value across the entire study period. Detection probabilities of frogs increased with survey effort and were higher in fall than in spring, but varied over time within sampling periods (Table 2). In both spring and fall, detection peaked when some but not all pools contained water (survey-specific water availability = 0.77; Fig 2). Although estimated abundance of adult frogs varied across seasons and years (Fig 3), there was no evidence of a systematic increase or decrease over time (slope = 6.2 ± 5.71 adult frogs/season, *P* = 0.29). Abundance did, however, vary with regional precipitation over the 16-year study period (Fig 4).

**Table 2:** Parameter estimates and 95% confidence intervals from a model describing abundance of adult leopard frogs in the Rincon Mountains, Arizona, USA between 1996 and 2011.

Parameter	Variable	Estimate	95% CI
Initial abundance (*λ*_1_) ^a^	Elevation ^b^	1.30	0.76–1.83
	Elevation^2^	-1.30	−1.66–−0.93
Recruitment (γ)	Season ^c^	-2.95	−3.71–−2.19
	Seasonal water availability	0.76	0.36–1.17
	No. pools/complex ^b^	0.65	0.60–0.71
	Connectivity (*S*_*i*_) ^b^	-0.15	−0.23–−0.06
Apparent survival (ω)	Season ^c^	2.31	1.61–3.00
	Seasonal water availability	3.61	2.69–4.53
Detection probability (*p*)	Effort	1.13	0.75–1.50
	Survey-specific water availability	9.01	7.69–10.34
	Survey-specific water availability^2^	-5.83	−6.80–−4.86
	Season ^d^	-1.34	−1.51–−1.18
	Day ^b^ ^,^ ^e^	-0.20	−0.29–−0.11
	Day^2^	0.03	−0.07–0.13
	Season*Day	0.20	0.06–0.33
	Season*Day^2^	-0.27	−0.40–−0.14

Parameter estimates for initial abundance and recruitment are on a log scale, and parameter estimates for apparent survival and detection probability are on a logit scale.

^a^ Log-transformed number of pools per complex was used as an offset for abundance in the first season.

^b^ Values were standardized relative to their means and standard deviations.

^c^ Indicator variable with summer = 1, winter = 0.

^d^ Indicator variable with spring = 1, fall = 0.

^e^ Day number of sampling period.

## Discussion

Spatial and temporal dynamics of many amphibian populations are influenced by changes in the distribution and availability of water, as most species require surface water or moist environments for one or more life stages [42]. Consequently, water availability has been used to explain variation in distribution and abundance (e.g., [43–45]) as well as overall rates of population change [46–47]. Although useful to describe dynamics at the population scale, distribution and abundance reflect a complex suite of interrelated individual-based demographic processes, including reproduction, growth, survival, and movements. Evaluating demographic rates independently allows us to better understand the mechanisms by which variation in hydrologic conditions govern the changes we observe in population-scale parameters.

Because amphibians have complex life cycles and different life stages frequently inhabit different environments [42], [48], hydrologic conditions may affect life stages differentially. Many previous studies have focused on the effects of hydrologic conditions on egg and larval stages because these aquatic life stages are often assumed to dictate population dynamics of amphibians [49–51]. Indeed, the number of metamorphosing larvae governs variation in population growth rates for many amphibian species [52–53] and varies frequently with hydrologic conditions. Specifically, the number of metamorphosing larvae in many amphibian populations can increase with higher precipitation or longer hydroperiods (e.g., [54–56]) and may approach zero in response to drought conditions [29], [57]. Although we characterized recruitment of adults rather than larvae or juveniles which is more common in studies of anurans, we found that recruitment of lowland leopard frogs increased as the amount of surface water available in bedrock-lined pools increased (Fig 1), reflecting an increase in this key habitat feature required by earlier life stages.

Historically, survival of adult amphibians has been thought to influence population dynamics less than recruitment, although recent empirical and theoretical studies have challenged this idea [48], [50], [53]. In many amphibian populations, survival of adults can remain relatively constant over time or can vary with habitat quality [50], [58–59]; few studies have explored relationships between adult survival and hydrologic conditions. In colder climates, survival often decreases as winter severity increases [46]. In warmer climates, however, relationships between survival and hydrologic conditions can be more complex. For example, survival of adult northern dusky salamanders (*Desmognathus fuscus*) in the eastern U.S. was unaffected by drought, likely because individuals were able to move to underground refugia where humidity was high [58]. In contrast, survival of adult African pig-nosed frogs (*Hemisus marmoratus*) in Ivory Coast declined in years with low rainfall, probably due to physiological stress or reductions in food resources [60]. We found that survival of adult lowland leopard frogs decreased as water availability decreased, perhaps reflecting the limited number of moist refugia available in these arid environments during periods of summer drought.

Although plainly important, water availability is not the only factor influencing survival and recruitment in lowland leopard frogs. Survival was higher during summer than during winter, similar to patterns observed elsewhere in their range [21]. Low water temperatures during winter have potential to decrease survival of frogs via direct and indirect mechanisms. Bd is distributed widely throughout amphibian populations in southern Arizona, including populations of lowland leopard frogs and canyon treefrogs (*Hyla arenicolor*) in the area we studied [26–28], [61]. In this region, infection rates increase as water temperature decreases [62]. Spatial or temporal variation in disease prevalence could explain some variation in abundance of leopard frogs by affecting survival, but prevalence of Bd has not been monitored rigorously in this region and little is known about winter survival in populations where Bd is not present, as may have been true historically.

Our estimates of adult recruitment potentially confound two processes occurring within and among populations: somatic growth and immigration. If a large proportion of recruits transitioned from juvenile to adult stage classes locally, we would expect a positive association between recruitment and the number of pools in each complex because an increase in the number of pools reflects an increase in the amount of habitat available to young frogs. If, however, recruitment was driven primarily by one-way movements of adult frogs between complexes (i.e., immigration), we would expect a positive relationship between recruitment and connectivity because individuals would be more likely to move into a complex in close proximity to other complexes. We found that recruitment rates were higher in complexes with more pools and in complexes that were more isolated, suggesting that growth and transition of juveniles into adults within a pool complex was the primary source of recruits whereas movement of adults between complexes was a relatively small source of recruits, at least in the canyons we studied. The limited and scattered distribution of water sources in these arid mountain canyons may explain, at least in part, why our findings seem to contradict recent studies that suggest amphibians are not as dispersal-limited as once assumed [63]. The measure of potential connectivity that we used to assess movement probabilities, however, was based on a limited number of marked individuals in only one year. Future studies that investigate connectivity more rigorously will allow us to evaluate better the frequency with which lowland leopard frogs make these inter-complex movements.

Like many amphibians, abundance of lowland leopard frogs in the populations we studied varied appreciably over time and in response to variation in hydrological conditions, even when these conditions were measured coarsely at a regional scale (Figs 3 and 4). In general, abundance of lowland leopard frogs in the populations we studied was similar to that of populations in other arid systems [32] but lower than many populations that inhabit lentic sites or streams with perennial flow [21], [27]. Survival rates were lower than those of other species of leopard frogs in Arizona that can live for 4–10 years after metamorphosis (e.g., *L*. *chiricahuensis*, *L*. *tarahumarae* [64–66]), but were comparable to *L*. *onca* (0.90), a threatened species with a limited distribution in northwestern Arizona and southern Nevada that is related closely to lowland leopard frogs [67–68]. Although low survival rates could be a proximate explanation for low abundances, ultimately aquatic habitat for lowland leopard frogs in the Sky Island region has been reduced markedly during the last century [16], [19], which in turn has reduced distributions and abundances of many aquatic organisms at a regional scale [20–23].

## Conclusions

Understanding changes in the availability of surface water is integral to understanding population dynamics of lowland leopard frogs, as even coarse measures of hydrologic conditions indicate strong associations between surface water and both recruitment and survival (Fig 1). Although abundance of lowland leopard frogs fluctuated with availability of surface water, populations have not declined precipitously as have other amphibian populations in the western U.S., particularly those associated with high prevalence of Bd (e.g., [69–70]). If, however, availability of surface water in these mountain canyons decreases, so might populations of leopard frogs and other aquatic vertebrates.

Climate change is a serious threat to aquatic obligates in this arid region because of anticipated increases in temperatures and decreases in precipitation, particularly during winter [12–13]. Although changes in climate are likely to reduce the amount of surface water available for leopard frogs and other aquatic species that rely on intermittent mountain streams, predicting accurately the volume of water in a particular canyon based on precipitation models alone will be challenging. In this system, water availability is especially dynamic because (1) pools are often recharged by both surface runoff and groundwater sources, (2) local geologic structure can affect rates of flow or water retention, and (3) sedimentation events associated with large wildfires, which are believed to be increasing in frequency with climate change, can alter drastically total pool capacity [14], [19], [71]. Despite the inherently dynamic nature of surface water in these environments, systematic and long-term declines in the availability of aquatic resources due to climate change have the potential to fundamentally change the distribution and amount of habitat for species that inhabit these isolated aquatic environments. Long-term conservation efforts for lowland leopard frogs and other aquatic species that inhabit wet areas in desert ecosystems may depend ultimately on a more complete understanding of processes that govern the dynamics of these water sources. Similar to historical changes observed in valley-bottom streams, streams in mountain canyons could potentially transition from intermittent to ephemeral flow, which could reduce recruitment and survival within, and connectivity among, populations of lowland leopard frogs and ultimately reduce aquatic biodiversity in these environments.
